# The Role of Nuclear-Encoded Mitochondrial tRNA Charging Enzymes in Human Inherited Disease

**DOI:** 10.3390/genes13122319

**Published:** 2022-12-09

**Authors:** Christina Del Greco, Anthony Antonellis

**Affiliations:** 1 Department of Human Genetics, University of Michigan Medical School, Ann Arbor, MI 48109, USA; 2 Department of Neurology, University of Michigan Medical School, Ann Arbor, MI 48109, USA

**Keywords:** protein synthesis, mitochondrial biology, aminoacyl-tRNA synthetases, neurological disease, tRNA biology

## Abstract

Aminoacyl-tRNA synthetases (ARSs) are highly conserved essential enzymes that charge tRNA with cognate amino acids—the first step of protein synthesis. Of the 37 nuclear-encoded human ARS genes, 17 encode enzymes are exclusively targeted to the mitochondria (mt-ARSs). Mutations in nuclear mt-ARS genes are associated with rare, recessive human diseases with a broad range of clinical phenotypes. While the hypothesized disease mechanism is a loss-of-function effect, there is significant clinical heterogeneity among patients that have mutations in different mt-ARS genes and also among patients that have mutations in the same mt-ARS gene. This observation suggests that additional factors are involved in disease etiology. In this review, we present our current understanding of diseases caused by mutations in the genes encoding mt-ARSs and propose explanations for the observed clinical heterogeneity.

## 1. Aminoacyl-tRNA Synthetases and the Mitochondria

Aminoacyl-tRNA synthetases (ARSs) are essential, highly conserved enzymes that ligate tRNA molecules to cognate amino acids, which is the first step of protein synthesis [1,2]. The human nuclear genome encodes 37 ARSs: 18 charge tRNA in the cytoplasm, 17 charge tRNA in the mitochondria, and 2 function in both compartments (specifically, glycyl-tRNA synthetase and lysyl-tRNA synthetase) by encoding two separate protein isoforms [1]. ARS-encoding genes are named by the single-letter code of the associated amino acid, followed by ‘ARS’ (e.g., *AARS* for alanyl-tRNA synthetase). Genes encoding ARSs that function specifically in the cytoplasm (or that encode bifunctional ARSs) are noted with a 1 (e.g., *AARS1*), while genes encoding ARSs that function exclusively in the mitochondria are noted with a 2 (e.g., *AARS2*).

**Figure 1:**
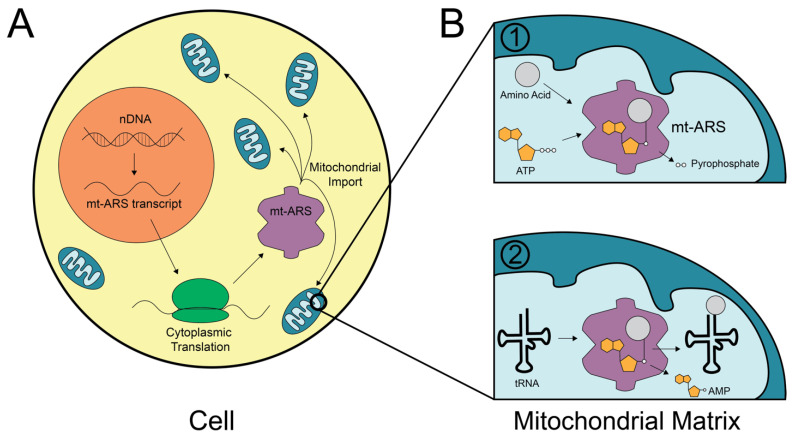
Mitochondrial aminoacyl-tRNA synthetases (mt-ARSs). (**A**) mt-ARSs are encoded by nuclear DNA (nDNA) and translated in the cytoplasm before being imported into the mitochondria. (**B**) mt-ARSs charge tRNA with corresponding amino acids in a two-step reaction (individual steps indicated by ‘1’ and ‘2’) in which the amino acid is first activated with ATP and then transferred to the tRNA molecule.

To perform aminoacylation in the mitochondria, mitochondrial ARSs (mt-ARSs) must be transcribed in the nucleus, translated in the cytoplasm, and imported into the mitochondria (Figure 1A). Mt-ARSs and cytoplasmic ARSs function via a two-step reaction in which a specific amino acid is activated by the ARS using a molecule of ATP, resulting in an aminoacyl adenylate intermediate. Next, the ARS binds to the appropriate tRNA molecule, most often (but not always) via an anticodon recognition domain. Finally, the amino acid is transferred to the acceptor stem, and the charged tRNA is delivered to the protein synthesis machinery (Figure 1B) [3,4]; all of these steps are essential for enzyme function, although there are certain cases where the order of the steps differ. Of note, mitochondrial glutaminyl-tRNA molecules do not have a dedicated mt-ARS; rather, glutamine aminoacylation occurs via the transamidation of glutamic acid. Here, mitochondrial glutamyl-tRNA synthetase (EARS2) aminoacylates tRNA^Gln^ as Glu-tRNA^Gln^. Next, the GatCAB complex (composed of three subunits encoded by *QRSL1*, *GATB*, and *GATC*) converts glutamic acid into glutamine [5].

The primary function of the mitochondria, known as the “powerhouse of the cell”, is to generate energy for cells via the production of ATP using oxidative phosphorylation [6]. This pathway uses FADH_2_ and NADH—generated by processing glucose through glycolysis and the tricarboxylic acid cycle—to generate ATP via the production of a proton gradient created by the oxidative phosphorylation complexes [7]. The mitochondrial genome encodes thirteen proteins, all of which are components of this pathway and are essential for oxidative phosphorylation [6]. The mitochondrial genome also encodes ribosomal RNA subunits and a full set of transfer RNAs, which are charged by mt-ARSs [8,9]. Additionally, mitochondria have secondary functions, including (i) the generation of reactive oxygen species, (ii) the regulation of metabolites, (iii) iron metabolism and heme synthesis, (iv) the biosynthesis of pyrimidines and lipids, and (v) the regulation of the nuclear epigenome [10,11]. It is therefore interesting to consider that mutations in genes important for mitochondrial function may have impacts beyond affecting cellular respiration.

## 2. Human Inherited Diseases Associated with Mt-ARSs

Combined, mitochondrial diseases are the most common group of neuro-metabolic disorders [12]. Because mitochondria are dependent on both mitochondrial- and nuclear-encoded genes, mitochondrial disease can be caused by mutations in the mitochondrial DNA or by mutations in the nuclear genome [13]. Mitochondrial DNA mutations are inherited maternally, and the associated diseases are often complicated by mitochondrial heteroplasmy, which arises due to the fact that an individual cell may have thousands of mitochondria, each containing 2–10 copies of mitochondrial DNA [13]. Heteroplasmy occurs when a cell has a mixed population of wild-type and mutant mitochondrial DNA, with more severe phenotypes typically associated with a higher percentage of mutant compared with wild-type [14]. Nuclear DNA encodes over 1000 mitochondrial-localized proteins [15], and while the majority of variants in nuclear-encoded mitochondrial genes are inherited in a recessive manner, there are some cases of dominantly inherited mitochondrial disease, such as paragangliomas associated with mutations in *SDHC* (succinate dehydrogenase complex subunit C) [13,16]. Additionally, some phenotypes can be inherited in both dominant and recessive fashions, such as optic atrophy caused by variants in *SSBP1* (single-stranded DNA-binding protein 1) [13,17]. Mitochondrial disease often presents in tissues with high energy demands, including the central nervous system, the cardiovascular system, and the musculoskeletal system, among other tissues [18,19]. Mitochondrial disease is also often associated with diabetes, along with other endocrine disorders [20]. Overall, mitochondrial disease is highly heterogeneous, and clinical phenotypes vary widely depending on which gene is affected.

Given their essential role in the translation of mitochondrial-encoded proteins, it is not surprising that all 17 mt-ARSs have been implicated in human disease [21]. Biallelic variants in genes encoding mt-ARSs are associated with a broad range of clinical phenotypes affecting organ systems with high energy requirements (Table 1) [21]. Many mt-ARSs are associated with central nervous system phenotypes, including encephalopathies and leukoencephalopathies (e.g., *DARS2* [22]) [23]. Another commonly affected tissue is the heart, and patients with recessive mt-ARS-associated disease often present with cardiomyopathy (e.g., *AARS2* [24]). Clinical phenotypes are often gene- and variant-specific, and they are highly heterogeneous depending on what gene is mutated. Thus far, there have been no cases of dominantly inherited mt-ARS-related disease. It is hypothesized that mt-ARS-associated disease is caused by a loss-of-function effect that severely reduces enzyme function and therefore impairs mitochondrial protein synthesis; it is important to note that a total loss-of-function would be incompatible with life. However, the diverse roles of mitochondria raise the possibility that defects in mitochondrial translation caused by mt-ARS variants will affect not only oxidative phosphorylation but also secondary mitochondrial functions, causing additional stress on susceptible tissues.

This review addresses outstanding questions related to the clinical heterogeneity of mt-ARS-associated human diseases. First, a simple impairment to mitochondrial protein synthesis does not explain the variability in clinical phenotypes observed between patients with mutations in different mt-ARSs. Second, the reduced function of a specific mt-ARS does not explain how different variants in that mt-ARS can lead to highly variable clinical phenotypes. Third, clinical phenotypes associated with mt-ARSs do not directly align with clinical phenotypes associated with variants in their respective mitochondrial tRNA genes. Finally, there is evidence that variants in mt-ARSs may signal downstream cellular stress response pathways, which may contribute to disease phenotypes. All of these observations indicate that mt-ARS-associated diseases may arise due to multiple factors downstream of the mutated mt-ARS. Exploring these questions more deeply will provide a better understanding of how mt-ARS mutations cause human disease.

## 3. Clinical Heterogeneity among Patients with Mutations in Different Mt-ARSs

Since the prevailing hypothesis for the mechanism of mt-ARS-associated disease is a loss-of-function effect and, therefore, a downstream reduction in mitochondrial protein synthesis, one expectation might be that mt-ARS-associated phenotypes would be similar, regardless of which locus is mutated. However, some disease phenotypes appear to be specific to a particular mt-ARS and are not observed in patients with mutations in other mt-ARS genes. One example of an mt-ARS being associated with a unique clinical phenotype is mitochondrial tyrosyl-tRNA synthetase (*YARS2*), which is the only mt-ARS associated with a syndrome characterized by myopathy, lactic acidosis, and sideroblastic anemia (MLASA), which can variably occur along with pancreatic insufficiency [64,74]. *YARS2*-associated MLASA is heterogenous in terms of age of onset and severity; some patients experience infantile-onset MLASA that is fatal, while other patients experience adolescent-onset, progressive MLASA [75]. Another example of highly specific phenotypes associated with mt-ARSs is mitochondrial isoleucyl-tRNA synthetase (*IARS2*), which is associated with a condition characterized by cataracts, growth hormone deficiency, sensory neuropathy, sensorineural hearing loss, and skeletal dysplasia (CAGSSS) [37]. While CAGSSS is not the only phenotype associated with *IARS2*, other phenotypes are less common.

One possible explanation for these observations is that defects in a given mt-ARS differentially affect the translation of a specific subset of proteins due to the amino acid content [76]. The thirteen mitochondrial-encoded proteins all have different amino acid compositions; for example, MT-ATP6 has nearly three times the isoleucine content compared with that of MT-ATP8 (12.8% vs. 4.4% isoleucine, respectively). Tyrosine content in mitochondrial-encoded proteins ranges from 1% (MT-ATP6) to 6% (MT-ND6), and the most extreme example is valine content, which ranges from 1% (MT-ATP8) to nearly 18% (MT-ND6) [76]. One way to assess this would be to carefully examine and compare patients with mutations in mt-ARSs associated with high amino acid content in the mitochondrial proteome with patients with mutations in mt-ARSs associated with low amino-acid content. For example, the mitochondrial proteome consists of 17% leucine but only 1.6% arginine [76]; as a result, patients with pathogenic variants in *LARS2* may be expected to have a more severe disease that affects a broader panel of tissues compared with those of patients with pathogenic variants in *RARS2*. A second possibility, which will be discussed below, is that certain mt-ARSs may have secondary functions; in this situation, the combined loss of protein synthesis and secondary function could result in distinct phenotypes.

## 4. Clinical Heterogeneity among Patients with Mutations in the Same Mt-ARSs

In addition to clinical heterogeneity among patients with pathogenic variants in different mt-ARS loci, there are cases of diverse phenotypes associated with variants in the same mt-ARS. That is, certain variants in a given mt-ARS can lead to one clinical phenotype, while other variants can lead to a distinct second phenotype. One example of this is *AARS2*, or mitochondrial alanyl-tRNA synthetase. *AARS2* has been associated both with leukoencephalopathy (often in combination with ovarian failure) and separately with hypertrophic cardiomyopathy [24]. These clinical phenotypes are seemingly non-overlapping. That is, patients with *AARS2*-related cardiomyopathy have not been reported to have leukoencephalopathy, and those with leukoencephalopathy have not been reported to have cardiomyopathy; in a review of 48 patients, no patients had both cardiomyopathy and neurological conditions [26]. The age of onset of clinical phenotypes in *AARS2* patients is also highly variable, ranging from infancy to over 40 years of age, and there does not seem to be an association between specific phenotypes and the age of onset [26]. 

Another gene associated with an interesting spectrum of clinical phenotypes is mitochondrial seryl-tRNA synthetase (*SARS2*). Patients with *SARS2* variants present with (i) a progressive spastic paresis [53]; (ii) a syndrome characterized by hyperuricemia, pulmonary hypertension, renal failure in infancy, and alkalosis (HUPRA) that is typically lethal within the first few years of life [52]; or (iii) a syndrome that includes both neurological and HUPRA phenotypes [77,78,79]. Interestingly, HUPRA syndrome is exclusively associated with *SARS2*, providing another example of unique mt-ARS phenotype. 

It is unclear why certain mutations in a given synthetase, such as *AARS2* and *SARS2*, lead to clinically distinct phenotypes, especially when the hypothesized mechanism is reduced enzyme function; based on the common role in mitochondrial protein synthesis, one would hypothesize that severely reducing the function of any mt-ARS would result in a similar clinical phenotype. One explanation for the above observations is that disparate phenotypes are not actually clinically distinct, but rather that the reports are prone to ascertainment bias based on the expertise of the examining physician. For example, if a patient with *SARS2* variants primarily sees a neurologist, HUPRA syndrome may be missed if the phenotype is subtle. This explanation would remain in line with a severe reduction of enzyme function if the effect of different genotypes on overall mt-ARS function varies. A related explanation is that different mutations—and therefore different genotypes—may have different effects on protein function; for example, some mt-ARS variants might affect tRNA recognition, while others might alter catalytic activity or mitochondrial localization, leading to a genotype-dependent spectrum of properly charged tRNA in the mitochondria. Alternatively, some mt-ARSs have an editing domain (such as *AARS2*) that deacylates incorrectly charged amino acids. Thus far, no patients with *AARS2*-associated, adult-onset leukoencephalopathy have variants in the editing domain, but there have been such variants identified in patients with *AARS2*-associated, infant-onset cardiomyopathy, indicating that certain variants may differentially affect aminoacylation and/or editing [25,80]; interestingly, the effect of a variant in the editing domain might result in a phenotype similar to those of variants that increase the likelihood of a given mt-ARS charging the incorrect amino acid via an alternative mechanism (e.g., altering the structure of the amino acid binding pocket). Relatedly, it is possible that some variants result in stably expressed proteins, while others result in proteins that are degraded. In this case, the stable expression of a defective protein might allow some level of function that could modify the clinical phenotype.

## 5. Incongruence of Phenotypes Associated with Mt-ARSs and tRNA Pairs

Mutations in mitochondrial tRNAs are also associated with a broad range of human disease phenotypes [81]. Like pathogenic mt-DNA variants, mutations in mt-tRNA genes can display heteroplasmy, further complicating the effects these variants have on mitochondria function since wild-type and mutant copies can be present in each cell [81]. As a result, the ratio of functional to non-functional mitochondria might vary significantly between patients with the same mt-tRNA mutation, which could lead to differential phenotypic effects. Clinical phenotypes associated with mt-tRNA genes include mitochondrial myopathy, encephalopathy, and stroke-like episodes (MELAS); maternally inherited diabetes and deafness (MIDD); Leigh syndrome; epilepsy; cardiomyopathy; and ataxia [21,81]. Interestingly, the phenotypes associated with mutant mt-ARSs do not always correspond with the phenotypes associated with mutated mt-tRNAs for the same amino acid. In general, mutations in mt-tRNAs have a more global effect on tissues than that of mutations in mt-ARSs [21].

One example of this incongruence is mitochondrial leucyl-tRNA synthetase (*LARS2*), which is associated with Perrault syndrome [40], a condition that affects the nervous system (leading to sensorineural hearing loss) and the ovaries (leading to premature ovarian failure). Indeed, these two tissue types are typically the only ones affected in patients with pathogenic *LARS2* variants. *LARS2* has also been associated with HLASA (hydrops, lactic acidosis, and sideroblastic anemia), which is another rare phenotype unique to *LARS2* [41,43]. In contrast, mt-tRNA^Leu^ mutations are associated with a broader array of clinical phenotypes. Mt-tRNA^Leu^ was first linked to MELAS [12] but has since been associated with various conditions, including diabetes mellitus and deafness [82], Kearns–Sayre syndrome [83], cardiomyopathy [84], and renal disease [85]. *YARS2* is another example of this incongruence; as previously discussed, *YARS2* is only associated with the MLASA phenotype. Mt-tRNA^Tyr^ mutations, however, have been associated with exercise intolerance [86], chronic progressive external ophthalmoplegia (CPEO) with myopathy [87], and focal segmental glomerulosclerosis (FSGS) and dilated cardiomyopathy [88]. While exercise intolerance and myopathy are somewhat consistent with an effect on skeletal muscle shared with MLASA, CPEO and FSGS affect two distinct tissue types—the ocular system and kidneys, respectively—that are not affected by the variants in *YARS2*. 

There are two likely explanations for the observation that mutations in mt-tRNAs do not cause the same disease phenotypes as mutations in the corresponding mt-ARS. The first is that certain mutations in a given mt-ARS could lead to similar phenotypes associated with corresponding mt-tRNA mutations, and that these patients simply have not yet been identified. Alternatively, it is possible that a lack of a particular charged mt-tRNA leads to different cellular effects than that of deficits in the total amount of that mt-tRNA. For example, mutations in mt-tRNAs might not impact tRNA charging but might instead cause decreased tRNA binding with the ribosome or other translation factors, leading to a different phenotype than that of depletions of charged mt-tRNAs. In that case, some undefined mechanism may compensate for insufficient mt-ARSs or mt-tRNAs may have other cellular functions even when uncharged that are lost when mt-tRNAs are mutated.

## 6. Potential Role of Non-Canonical Mt-ARS Functions in Disease Phenotypes

There is an increasing body of work suggesting that cytoplasmic and mitochondrial ARSs have additional cellular functions aside from aminoacylation [89]. For example, cytoplasmic threonyl-tRNA synthetase (TARS1) has documented roles in angiogenesis [90] and translation initiation [91], and cytoplasmic seryl-tRNA synthetase (SARS1) contributes to regulating angiogenesis [92]. Additionally, many synthetases have nuclear localization signals and play roles in transcriptional regulation [3]. Furthermore, many cytoplasmic synthetases also participate in the multi-synthetase complex, which includes nine synthetases and regulates canonical and non-canonical ARS functions [93,94]. 

The majority of described non-canonical functions have been for cytoplasmic synthetases; however, it is possible that mt-ARSs have non-canonical functions. Evidence from experiments that use centrifugation to separate soluble and membrane mitochondrial fractions has shown that certain mt-ARSs (DARS2, RARS2, and the bifunctional KARS1) localize to distinct parts of the mitochondria, suggesting that they have non-canonical functions that are mitochondrial-compartment-specific [95]. Additionally, given the fact that the mitochondria perform functions aside from oxidative phosphorylation, it is possible that mt-ARSs contribute to these roles. For example, FARS2 and WARS2 have pro-angiogenic functions [96,97], and TARS2 is required for threonine-dependent mTORC1 activation [98]. Additionally, recent studies of the METTL8 protein, which is a methyltransferase that modifies mitochondrial tRNAs with 3-methylcytidine at position 32 (m^3^C32) on mt-tRNA^Thr^ and mt-tRNA^Ser^ (UCN), revealed an interaction with mitochondrial seryl-tRNA synthetase (SARS2) via the immunoprecipitation of METTL8; interestingly, SARS2 was the only synthetase identified in these experiments, and the interaction was specific to METTL8 rather than to other methyltransferase proteins like METTL6 [99,100]. METTL8 is also part of a nuclear RNA-binding complex that may methylate mRNAs, but it has multiple alternatively spliced transcripts that coordinate the localization of METTL8 to the mitochondria for m^3^C32 modifications [100]. It has been hypothesized that these m^3^C32 modifications are necessary for proper tRNA folding, and there is evidence from overexpression experiments that the dosage of SARS2 can partially modulate the m^3^C32 modification activity of METTL8 [99,100]. While evidence for non-canonical functions has only been described for a fraction of the mt-ARSs, it is clear that they play essential roles in different cellular functions, and additional research is needed to determine if other mt-ARSs have non-canonical functions that explain the clinical heterogeneity of mt-ARS-associated human disease.

## 7. Downstream Consequences of Mt-ARS Variants on Cellular Stress Responses

Reduced function of ARSs has been linked to cellular stress responses, specifically the integrated stress response (ISR) and the unfolded protein response (UPR), leading to the hypothesis that these pathways contribute to the clinical phenotypes associated with these ARSs. The ISR controls the protein synthesis in stress conditions signaled from the endoplasmic reticulum and the cytoplasm [101]. In response to stress signals, the ISR represses translation while specifically increasing translation of mRNAs that are capable of responding to stress; if the cellular stress cannot be resolved, this process can trigger apoptosis [102]. The ISR can be activated by different kinases, depending on the type of stress response; mTORC1 is activated in mitochondrial stress and signals the ISR, the mitochondrial UPR, and the one-carbon metabolism cycle [103]. The UPR responds to misfolded proteins and other stressors like oxidative stress and hypoxia to maintain mitochondrial protein homeostasis by upregulating the transcription of mitochondrial chaperone proteins and proteases, while the one-carbon metabolism pathway regulates biosynthetic processes, including amino-acid homeostasis [104,105].

Variants in the bifunctional glycyl-tRNA synthetase (*GARS1*) have been implicated in activating the ISR, and knockdown of the ISR has been shown to modulate dominantly inherited *GARS1*-related phenotypes [106]. Mitochondrial ARSs have also been connected to cellular stress responses. Mitochondrial aspartyl-tRNA synthetase (*DARS2*) has been linked to the mitochondrial UPR, as demonstrated by studies in *DARS2* conditional knockout mice [107]. The mutant mice developed cardiomyopathy, and a western blot analysis of the stress response transcription factors ATF5 and CHOP confirmed UPR upregulation [107]. Additionally, mice homozygous for a *WARS2* mutation showed ISR upregulation in western blots for ATF4 [108]. In sum, it is possible that the induction of cellular stress responses contributes to the observed clinical phenotypes in mt-ARS-associated disease.

Interestingly, in the mouse studies mentioned above, *DARS2*-associated activation of the UPR was tissue-specific; according to western blot data, the UPR was strongly activated in cardiac tissue but not in skeletal muscle, despite a 60–80% decrease in mitochondrial oxidative phosphorylation complex activity using in-gel activity assays [107]. Similarly, western blot data revealed that the ISR activation observed in the *WARS2* mutant mice appeared heart-specific and did not affect kidney, skeletal muscle, and liver tissues [108]. These data would indicate that (a) certain tissues are more affected by pathogenic mt-ARS variants and/or that (b) certain tissues more readily activate cellular stress response signaling. Both of these possibilities are consistent with the observation of tissue-specific clinical phenotypes for mt-ARSs. Because cellular stress responses are programmed to activate in instances of tRNA depletion, it is unsurprising that stress response activation would be observed in cases of mt-ARS-related disease. There are additional stress response pathways such as the heat shock response (HSR), which modulates cellular protein folding and degradation in response to stresses including exposure to oxidants, that could also play a role in disease etiology [109]. Further investigation is necessary to determine which, if any, cellular stress responses are activated in each mt-ARS-related disease.

## 8. Remaining Questions on the Molecular Mechanisms of Mt-ARS-Associated Inherited Disease

**Figure 2:**
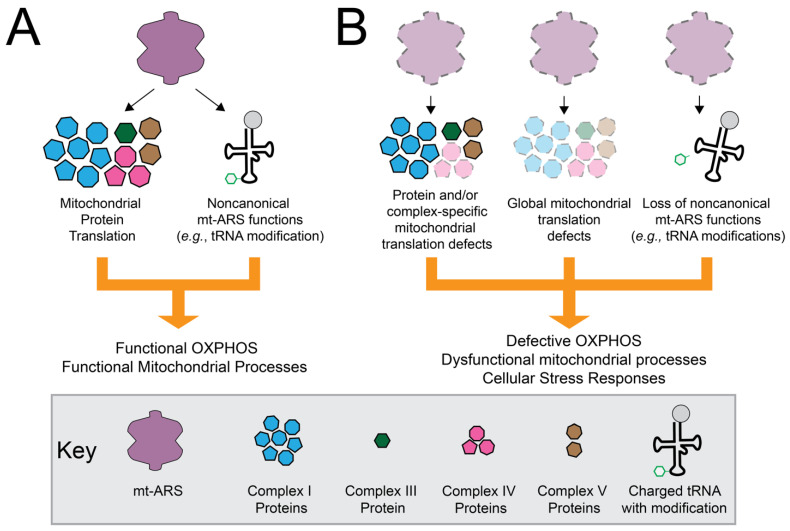
Potential mechanisms by which mt-ARSs may cause inherited disease. (**A**) mt-ARSs charge tRNA in the mitochondria and may also perform secondary functions important for supporting mitochondrial protein synthesis and mitochondrial function (e.g., tRNA modifications). (**B**) Pathogenic mt-ARS variants could disrupt canonical and non-canonical functions, causing protein or complex-specific deficits in mitochondrial translation; global deficits in mitochondrial translation; and/or impaired non-canonical functions, which reduce overall mitochondrial function and potentially activate cellular stress pathways.

Several questions need to be addressed to fully understand the locus, allelic, and clinical heterogeneity and the molecular mechanisms of mt-ARS-associated inherited diseases. While we know that mt-ARSs perform tRNA aminoacylation and, potentially, additional non-canonical functions (Figure 2A), we are still left with questions regarding the pathogenic mechanism(s) that lead to clinical phenotypes (Figure 2B) and how to approach therapeutic development. Addressing these and other questions will improve the ability of clinicians to provide accurate diagnoses and prognoses and to explore therapeutic options for affected patient populations.

## 9. What Is the Full Range of Clinical Phenotypes Associated with Mt-ARS Disease?

As discussed throughout this review, the diseases associated with pathogenic mt-ARS variants display a wide range of clinical phenotypes, affecting the central nervous system, the cardiovascular system, the musculoskeletal system, and other systems [95]. However, despite a likely shared mechanism of reduced tRNA charging in the mitochondria, multiple observations suggest that additional factors are at play in determining patient phenotypes. These observations include the following: (1) clinical phenotypes are often mt-ARS-specific; (2) clinical phenotypes are often variant- and genotype-dependent for a given mt-ARS; and (3) the clinical phenotypes associated with mt-ARSs do not always match the clinical phenotypes associated with variants in corresponding tRNA genes. Thus, the spectrum of clinical phenotypes associated with mutations in mt-ARSs is likely to expand. As additional pathogenic variants are identified, patient phenotypes should be carefully assessed toward fully annotating the complete spectrum of clinical phenotypes associated with these genes. Broadening and carefully defining this spectrum will provide the basis for research on the mechanisms that underlie tissue-specific and tissue-predominant phenotypes.

## 10. How Do Locus and Allelic Heterogeneity Impact Clinical Heterogeneity?

Several examples were presented in this review where different mutations in the same mt-ARS cause distinct clinical phenotypes. One possibility that may explain this observation is that the varying output of each genotype leads to differential functional consequences that dictate phenotype specificity and severity. To address this, careful biochemical and cellular studies are needed to quantify the precise effect of each mt-ARS mutation on tRNA charging and mitochondrial function. Furthermore, massively parallel mutagenesis studies [110] aimed at identifying all loss-of-function mutations in mt-ARSs (and aimed at quantifying these loss-of-function effects) would expedite patient diagnosis and allow assessments of the effect of each allele and genotype on gene function. 

## 11. What Additional Functions Do Mt-ARSs Have in the Mitochondria?

As noted, evidence is mounting for the non-canonical functions of ARSs [89]. While much of this evidence is associated with cytoplasmic ARSs, there is a growing body of work demonstrating that mt-ARSs play additional roles in the mitochondria (e.g., SARS2 [99]). This has significant bearing on the downstream consequences of mutations in any given mt-ARS; while losing the function of any mt-ARSs would affect mitochondrial protein synthesis, it may also affect mt-ARS-specific non-canonical functions if the amino-acid residues impacted are important for those functions. For example, loss of *SARS2* would cause defects in mitochondrial protein synthesis and m^3^C32 tRNA modifications, but loss of function in another mt-ARS would likely leave m^3^C32 tRNA modification intact. Such observations could tease apart mt-ARS-specific clinical phenotypes and genotype-phenotype correlations. It is possible that this non-canonical role of SARS2, for example, contributes to the uniqueness of the HUPRA syndrome phenotype; given that HUPRA syndrome has only been associated with *SARS2*, it is possible that a loss of SARS2 function is not only leading to defects in mitochondrial translation due to a lack of charged tRNA^Ser^ but is also due to a lack of m^3^C32 on both mt-tRNA^Ser^ (UCN) and mt-tRNA^Thr^. Relatedly, it is possible that certain variants might affect only canonical aminoacylation activity and not non-canonical functions, and vice versa, which could contribute to variant-specific phenotypes. There are multiple mt-ARSs that contain protein domains that are potentially unrelated to canonical functions (e.g., DARS2 has a bacterial extension [111], and SARS2 and VARS2 contain C-terminal sequences that are uncharacterized [21]), and these domains are good candidates for identifying non-canonical functions. Thus far, the majority of mt-ARS variants tested have demonstrated loss-of-function effects; pathogenic variants that preserve aminoacylation function may also point toward effects on non-canonical functions. Overall, studies to identify potential secondary functions of mt-ARSs will be essential for fully understanding disease mechanisms. 

## 12. How Do Pathogenic Mt-ARS Variants Affect Cellular Physiology?

Downregulating cytoplasmic and mitochondrial translation has well-defined negative effects on cell biology. For example, cellular stress pathways, including the ISR [106,108,112] and UPR [107], are activated in an attempt to combat these translation defects, and if not resolved, apoptosis ensues. Thus far, stress response signaling has not been identified in all cases of mt-ARS-related disease. However, given that the severely reduced function of any mt-ARS would potentially lead to, for example, a buildup of uncharged tRNA in the mitochondria, a cellular stress response activation would be expected. 

It is also reasonable to hypothesize that other cell signaling pathways could be activated in the context of these pathogenic variants, especially when considering potential secondary functions of mt-ARSs. For example, tRNA modifications play a role in managing cellular stress, and mitochondrial tRNA-derived fragments (tRFs), which are small non-coding RNAs that are often regulated by tRNA modifications, also regulate cellular stress pathways [113]. If mt-ARSs such as SARS2 play a role in tRNA modifications, any regulatory pathways managed by such modifications would be disrupted. 

## 13. How Do We Develop Therapeutics for Patients with Mt-ARS-Related Diseases?

Current therapeutic approaches for mt-ARS-associated diseases include treatments for general mitochondrial disease and/or the management of specific phenotypes; for example, in a case of *SARS2*-related HUPRA syndrome, the patient was treated with sildenafil for pulmonary hypertension, allopurinol for hyperuricemia, and α-lipoic acid and coenzyme Q10 for mitochondrial oxidative phosphorylation deficiencies [114]. While these drugs are treating the symptoms of HUPRA syndrome, they are not directly addressing the pathogenic mt-ARS variants. Amino acid supplementation has been used in cases of cytoplasmic ARS-related disease, as there is some evidence that supplementing the amino acid charged by the defective tRNA can improve clinical phenotypes [115]. Thus, it is possible that a similar approach could effectively treat patients with mt-ARS-associated disease. Additionally, in cases where at least one splice-site variant is involved in disease pathogenesis (e.g., *DARS2*), screens could be performed to identify chemical compounds that alter splicing patterns to support wild-type splicing [116]. In terms of future therapeutics, it is first important to determine exactly how each synthetase (and each variant within each synthetase) causes disease in order to optimize the development of effective treatments. 

It is also important to understand how each mutation and genotype affect downstream pathways, which may then be leveraged to develop therapeutics. For example, inhibiting the ISR in a *GARS1*-associated dominant disease reverses the phenotype in mouse models heterozygous for pathogenic *GARS1* variants (missense and in-frame deletion mutations) [106]. It remains to be seen if this is applicable to humans, applicable to all *GARS1* variants, and/or applicable to mutations in other synthetases. However, a better understanding of the relationship between defects in mt-ARSs and cellular stress responses could reveal promising therapeutic avenues.

## 14. Summary and Concluding Remarks

The literature on mt-ARS biology and related genetic diseases is growing rapidly. We are gaining a broader understanding of the complicated relationship between mt-ARSs and disease, which indicates that pathogenic mechanisms go beyond a “simple” loss-of-function effect. Additionally, emerging evidence suggests that mt-ARSs have non-canonical functions beyond tRNA charging. Thus, to fully understand the etiologies of mt-ARS-associated diseases, the following questions must be addressed: (1) What is the full range of clinical phenotypes associated with mt-ARS disease? (2) How do locus and allelic heterogeneity impact clinical heterogeneity? (3) What additional functions do mt-ARSs have in the mitochondria? (4) How do pathogenic mt-ARS variants affect cellular physiology? and (5) How do we develop therapeutics for patients with mt-ARS-related diseases? Addressing these questions will improve our understanding of mt-ARS-associated disease, improve mt-ARS patient diagnosis and prognosis, and broaden our understanding of the function of mt-ARSs and mitochondrial biology.

## Figures and Tables

**Table 1:** mt-ARS genes and associated clinical phenotypes. Acronyms not defined here (CAGSSS, HLASA, HUPRA, and MLASA) are defined in the body of the text.

Gene	Phenotype
*AARS2*	Infantile hypertrophic cardiomyopathy [24]; premature ovarian failure [25]; leukoencephalopathies [26]; ataxia [27]
*CARS2*	Alpers’ syndrome [28], progressive myoclonic epilepsy [29]
*DARS2*	LBSL (leukoencephalopathy with brainstem and spinal cord involvement and lactate elevation) [30]; hereditary spastic paraplegia [31]
*EARS2*	LTBL (leukoencephalopathy with thalamus and brainstem involvement and high lactate) [32]; COXPD12 (combined oxidative phosphorylation deficiency 12, including lactic acidosis and intellectual disability) [33]
*FARS2*	Alpers’ syndrome [28]; spastic paraplegia [34]; combined oxidative phosphorylation deficiency type 14 (developmental delay with elevated lactate, early-onset encephalopathy, liver failure, and hypotonia) [35]
*HARS2*	Perrault syndrome [36]
*IARS2*	CAGSSS [37]; Leigh syndrome [38]; West syndrome [39]
*LARS2*	Perrault syndrome [40]; HLASA [41]; deafness and ovarioleukodystrophy [42]; reversible myopathy, developmental delay, and lactic acidosis [43]
*MARS2*	Spastic ataxia [44]; COXPD25 (developmental delay, growth delay, and sensorineural hearing loss) [45]
*NARS2*	Alpers’ syndrome [28]; Leigh syndrome [38]; non-syndromic deafness and Leigh syndrome [46]; rapidly progressive intractable epilepsy and global brain atrophy [47]; mild intellectual disability and epilepsy [48]; myopathy, excessive fatigue, and ptosis [48]
*PARS2*	Alpers’ syndrome [28]; developmental delay with hypotonia, microcephaly, seizures, and cardiomyopathy [49]
*RARS2*	Pontocerebellar hypoplasia [50]; epileptic encephalopathy [51]
*SARS2*	HUPRA syndrome [52]; progressive spastic paresis [53]
*TARS2*	Mitochondrial encephalomyopathy [54]
*VARS2*	Mitochondrial encephalomyopathy [54]; encephalocardiomyopathy [55]; encephalopathy [56]; combined oxidative phosphorylation deficiency type 20 (developmental delay with microcephaly and seizures) [57]
*WARS2*	Infantile-onset leukoencephalopathy [58]; recessive intellectual disability [59]; mitochondrial encephalopathy [60]; levodopa-responsive infantile-onset parkinsonism [61]; hyperkinetic movement disorder [62]; dopa-responsive early-onset parkinsonism and progressive myoclonus ataxia [63]
*YARS2*	MLASA [64]
*GARS1*	Charcot-Marie-Tooth Type 2 [65]; spinal muscular atrophy [65]; systemic mitochondrial disease, including cardiomyopathy [66]
*KARS1*	Sensorineural hearing loss [67]; Charcot-Marie-Tooth disease, recessive intermediate [68]; optic neuropathy [69]; hypertrophic cardiomyopathy and mitochondrial complex deficiency [70]; microcephaly [71]; leukoencephalopathies [42]
GatCAB Complex	Lethal metabolic cardiomyopathy [72]; pediatric cardiomyopathy with early onset brain disease [73]; tachypnea, hypertrophic cardiomyopathy, adrenal insufficiency, hearing loss, and combined respiratory chain complex deficiencies [70]

## Data Availability

Not applicable.
